# Supplementary material for: Evaporated nanometer chalcogenide films for scalable high-performance complementary electronics
Source: Nat Commun. 2022 Oct 26;13:6372. doi: 10.1038/s41467-022-34119-6 (PMC9605968; doi:10.1038/s41467-022-34119-6)
Supplement: Supplementary file 1 — Supplementary information [file 41467_2022_34119_MOESM1_ESM.pdf]

Supplementary information

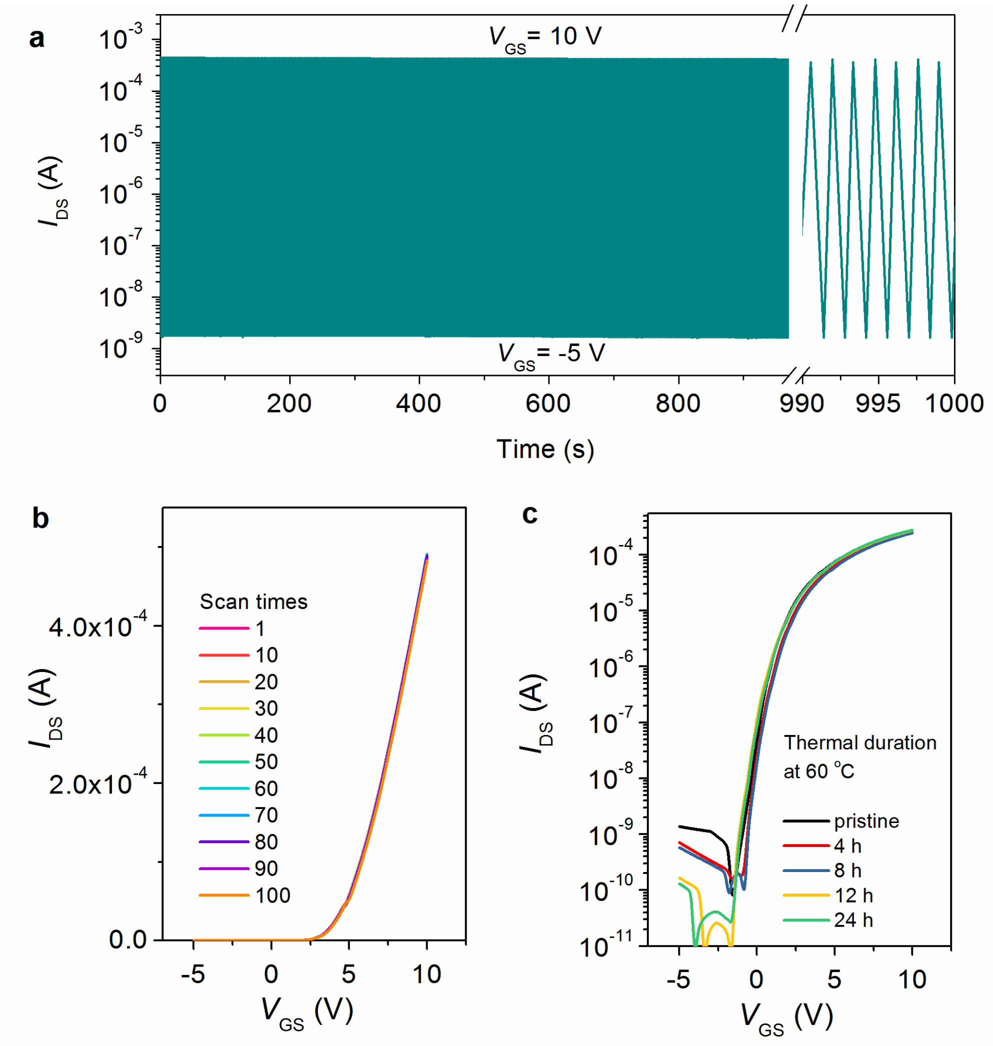

Supplementary Figure 1: Stability test of optimised Bi_2S_3 TFTs. a, Successive on/off switching test with 1400 cycles of one representative 250 °C-annealed Bi_2S_3 TFT. **b**, Consecutive transfer curve scan for 100 cycles (V_{DS} = 10 V). **c**, Transfer curves for the Bi_2S_3 TFT under thermal stress with different time (V_{DS} = 10 V).

The optimized 250 °C-Bi_2S_3 TFTs exhibit good operational stability with constant current response with clear on/off state under the fast voltage switching test. The negligible variation on transfer curve was also observed after continuous 100 cycling. The device also delivers stable operation under long-term thermal bias treatment at 60 °C.

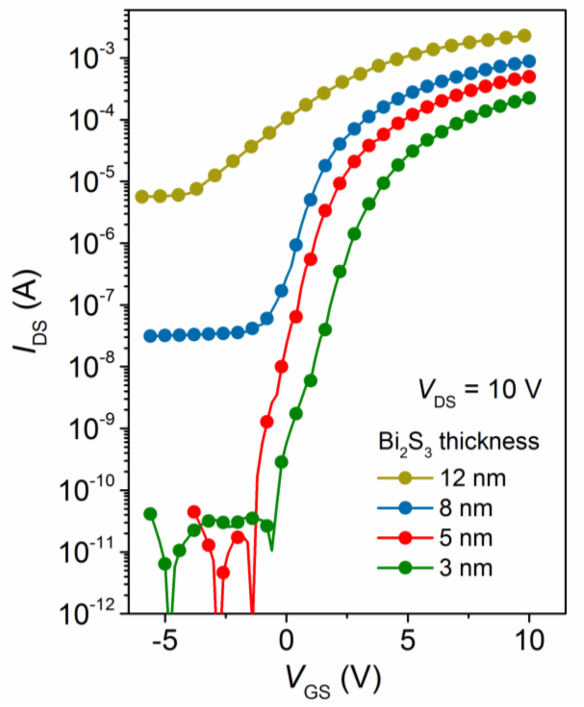

Supplementary Figure 2: Effect of channel thickness on the optimised Bi_2S_3 TFT performance. Transfer curves of 250 ^{o}C-Bi_2S_3 TFTs with different Bi_2S_3 channel thicknesses.

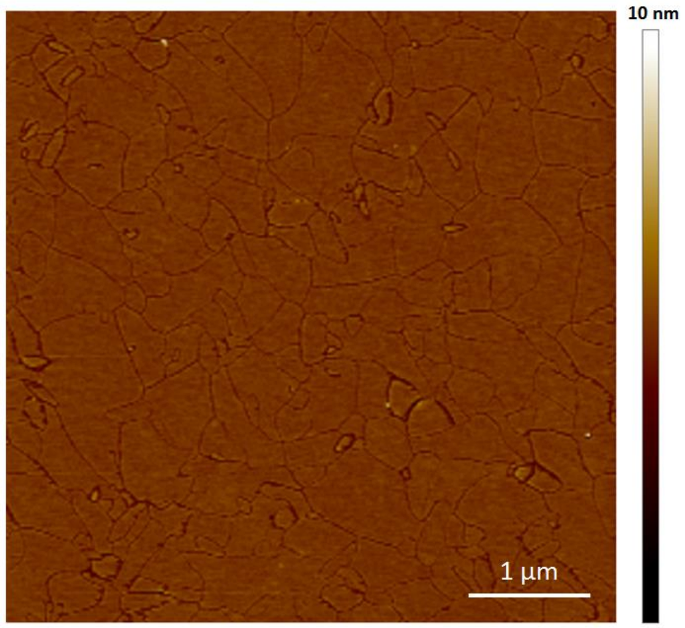

Supplementary Figure 3: Morphology of the optimised Bi_2S_3 thin film. AFM image of one 250 ℃-annealed Bi_2S_3 film.

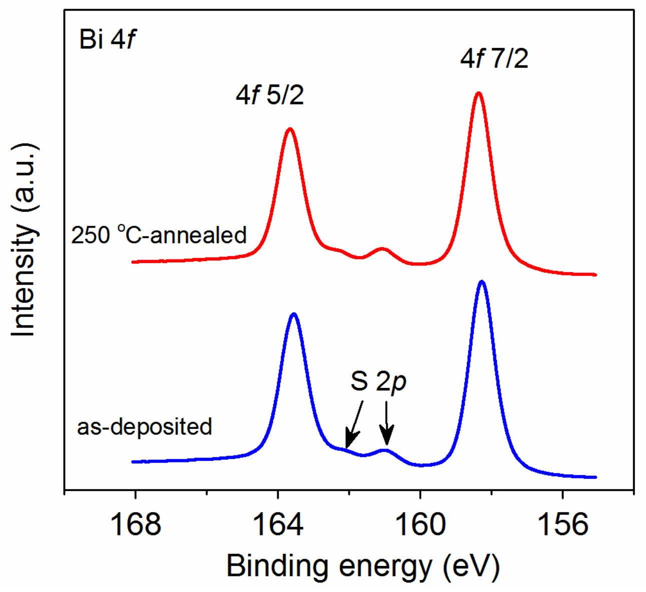

Supplementary Figure 4: XPS characterization of different Bi_2S_3 films. XPS Bi 4*f* spectra of as-deposited Bi_2S_3 thin film and the one annealed at 250 °C.

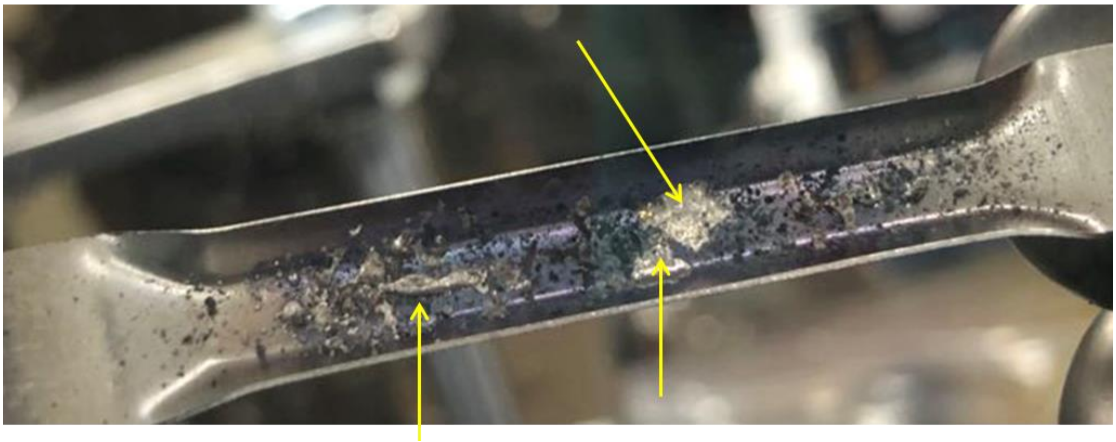

Supplementary Figure 5: Picture of tungsten boat after thermal evaporation. Bi_2S_3 power is aterrimus and the aggregate with silvery metallic luster is possible metallic Bi.

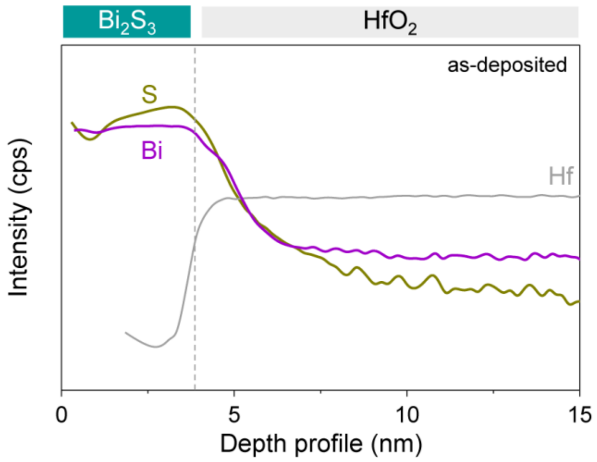

Supplementary Figure 6: Component analysis of as-deposited Bi_2S_3 thin film. SIMS spectra of the as-evaporated Bi_2S_3 thin film.

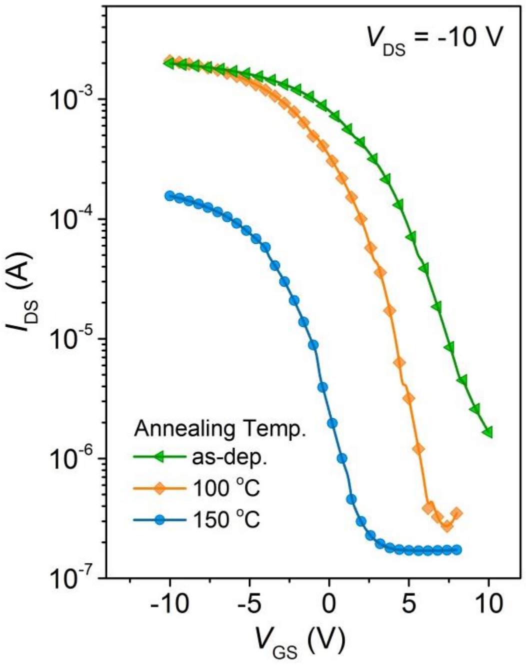

Supplementary Figure 7: Effect of postannealing on the Te TFT performance. Transfer curves of RT-evaporated Te TFTs as a function of postannealing temperature.
